# Supplementary material for: 3D atomic-scale metrology of strain relaxation and roughness in Gate-All-Around transistors via electron ptychography
Source: Nat Commun. 2026 Feb 23;17:3561. doi: 10.1038/s41467-026-69733-1 (PMC13087028; doi:10.1038/s41467-026-69733-1)
Supplement: Supplementary file 1 — Supplementary Information [file 41467_2026_69733_MOESM1_ESM.pdf]

Supplementary Information for 3D Atomic-Scale Metrology of Strain Relaxation and Roughness in Gate-All-Around (GAA) Transistors via Electron Ptychography

Shake Karapetyan[1], Steven E. Zeltmann[1,2], Glen Wilk[3], Ta-Kun Chen[4], Vincent D.-H. Hou[4] and David A. Muller[1,5*]

[1]School of Applied and Engineering Physics, Cornell University, Ithaca, NY, United States.

[2]Platform for the Accelerated Realization, Analysis, and Discovery of Interface Materials (PARADIM), Cornell University, Ithaca, NY, United States.

[3]Advanced Semiconductor Materials (ASM) America, Phoenix, AZ, United States.

[4]Corporate Analytical Laboratories, Taiwan Semiconductor Manufacturing Company, Hsinchu, Taiwan.

[5]Kavli Institute at Cornell for Nanoscale Science, Cornell University, Ithaca, NY, United States.

* Corresponding author: david.a.muller@cornell.edu

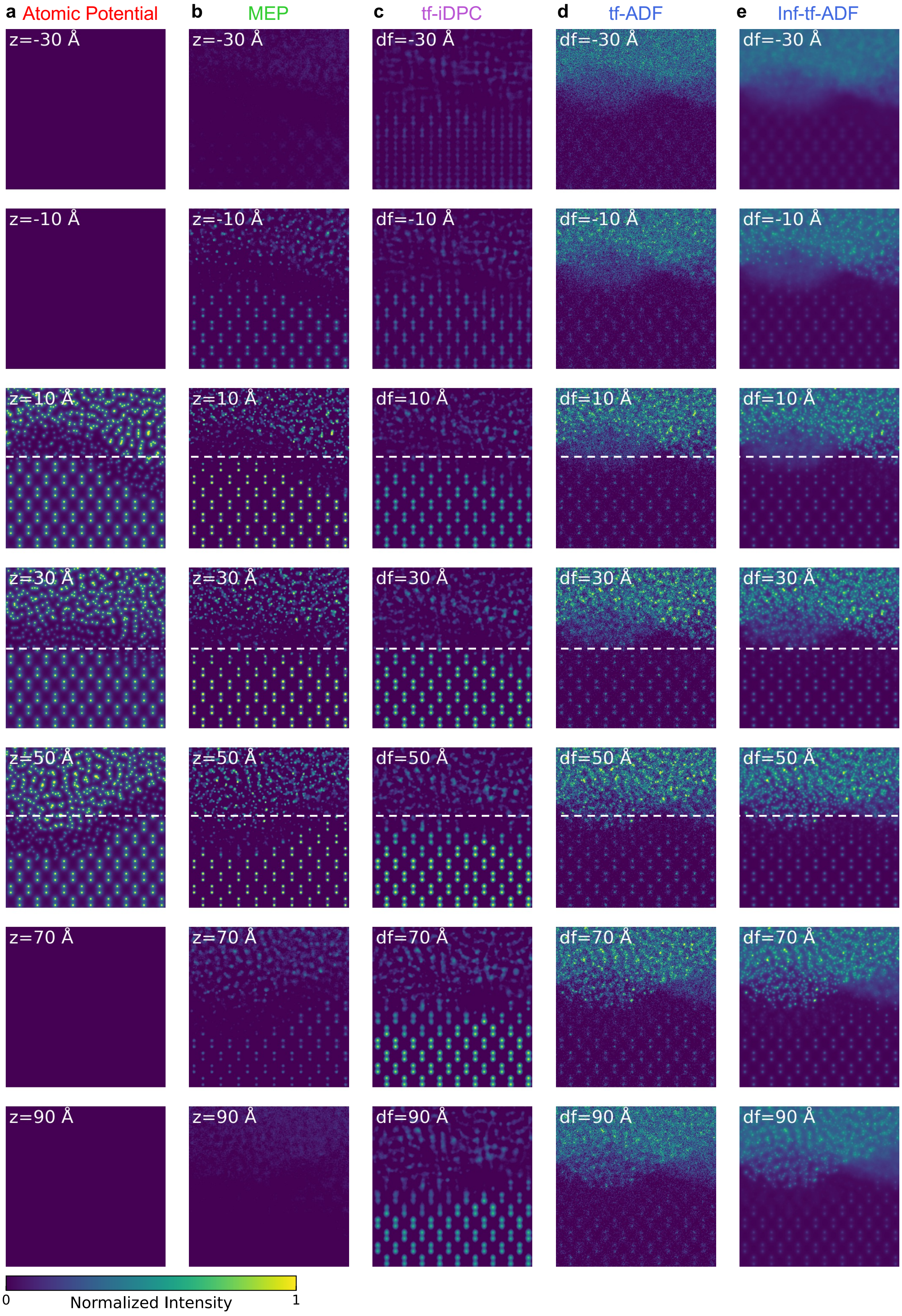

a Atomic Potential
b MEP
c tf-iDPC
d tf-ADF
e Inf-tf-ADF
z=-30 Å
z=-10 Å
z=10 Å
z=30 Å
z=50 Å
z=70 Å
z=90 Å
df=-30 Å
df=-10 Å
df=10 Å
df=30 Å
df=50 Å
df=70 Å
df=90 Å
0
Normalized Intensity
1

Supplementary Figure 1. Selected depth slices comparing depth-sectioning capabilities of MEP, tf-iDPC and tf-ADF imaging techniques for a c-Si/a-SiO_2/a-HfO_2 model interface. a Ground truth atomic potential depth slices, each 10 Å thick, with vacuum added before and after to match the total thickness of the simulations. **b** Corresponding depth slices of the MEP-reconstructed atomic potential, each 10 Å thick, accurately recovering the structure and roughness of the interface. **c** Corresponding tf-iDPC images from the stack, generated with a 10 Å defocus step, showing significant elongation of features in depth and loss of interface clarity. **d** tf-ADF images from the stack, also generated with a 10 Å defocus step, suffering from noise and poor depth resolution. **e** Corresponding infinite-dose (noise-free) tf-ADF images, highlighting the intrinsic elongation of features in depth even under ideal conditions. All images share a normalized intensity range from 0 to 1, scaled by the maximum value of their respective 3D stack. Spherical and chromatic aberrations are included in all datasets. Except for **e**, all having a total dose of 2.5×10^5 $e^-/Å^2$ per stack. The field of view is consistently 35 Å across all images. White dotted lines for z=10, 30, 50 Å are aligned with the topmost Si atom in the ground truth at the respective depths, highlighting the sinusoidal interface shape. Only the MEP reconstruction recovers the correct shape, depth position, and roughness of the interface.

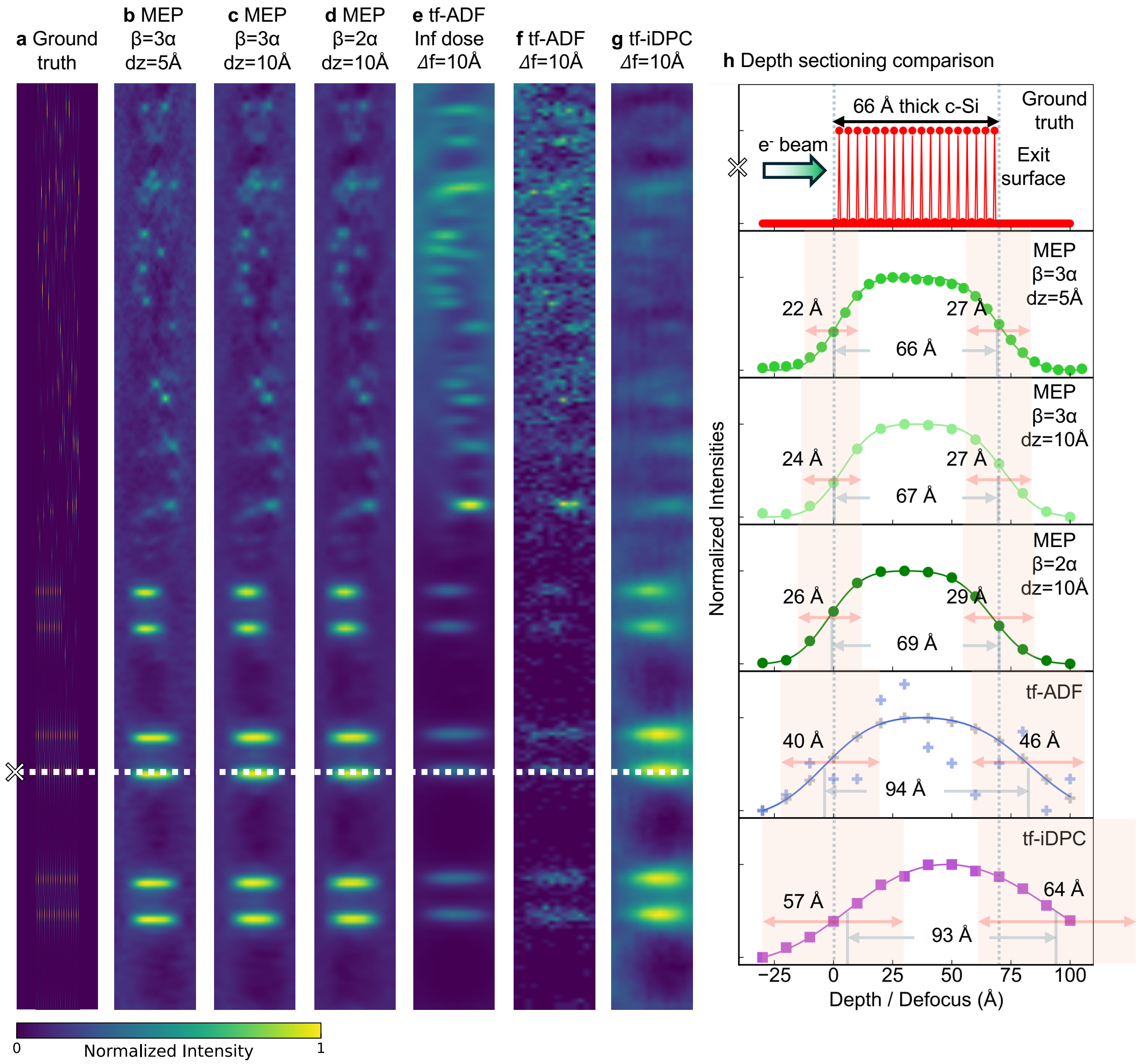

Supplementary Figure 2. Simulation of depth-sectioning performance for MEP for different collection and reconstruction parameters. **a** Ground-truth atomic potential used for the reconstructions shown in **b–g**. **b–d** MEP depth sections reconstructed using different detector collection angles (β = 3α or 2α) and slice spacings (dz = 5 Å or 10 Å). All MEP reconstructions recover the axial position and relative intensity of the 66 Å-thick Si column with minimal differences. This demonstrates that once the slice spacing satisfies the Nyquist criterion, reducing dz does not improve the achievable depth resolution. Furthermore, the similarity between the β = 3α and β = 2α reconstructions shows that increasing the collection angle does not improve resolution at a fixed dose (2.5×10^5 $e^-/Å^2$ here), because there little high-angle scattering signal is to be informative. **e–g** Corresponding depth sections for infinite- and finite-dose tf-ADF and tf-iDPC for reference. All images share a normalized intensity range from 0 to 1, scaled by the maximum value of their respective 3D stack. **h** (Top) Ground-truth column profile. (Middle three panels) Error-function fits for MEP reconstructions, showing consistent edge blurs of 22-29 Å (of

entrance and exit surfaces) and recovered column extents (66-69 Å) across all tested conditions. Thus, once dz meets Nyquist and β exceeds the range containing significant scattered intensity at the chosen dose, further refinement of either parameter does not improve depth resolution. (Bottom two panels) Fits for tf-ADF and tf-iDPC, which exhibit substantially larger edge broadening (40-64 Å) and apparent column widths (93-94 Å).

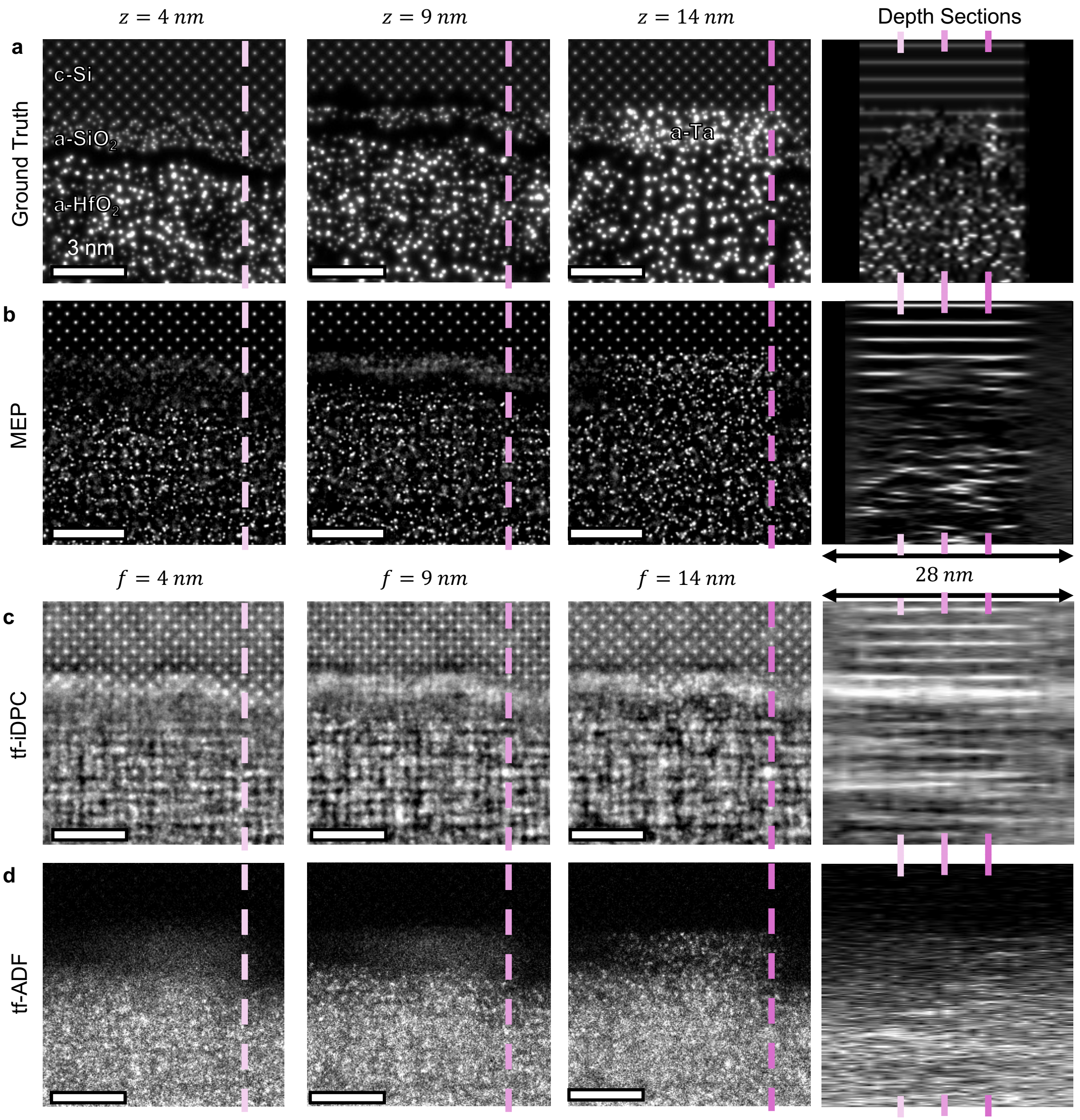

Supplementary Figure 3. Benchmarking the 3D imaging capabilities of MEP, tf-iDPC, and tf-ADF through simulations of a pMOS device structure. a Atomic potential of a section of a pMOS device structure with 1 nm depth blurring, shown at three depths. The model includes an intrusion defect filled with amorphous tantalum (Ta) (brighter region) at z = 14 nm. Dotted lines indicate the location along which the depth section is taken, shown in the rightmost panel. **b** MEP reconstruction atomic potential of the pMOS structure. The three panels correspond to the same depths as in **a**, with the intrusion defect resolved at z = 14 nm. Dotted lines indicate the depth section shown on the right. **c** Simulated tf-iDPC images at corresponding depths. The intrusion defect is difficult to resolve. **d** Simulated tf-ADF images at corresponding depths. While the

intrusion defect is visible, the lighter atoms are not. The total dose for MEP and tf-iDPC simulated stacks is 0.4 × 10^5 $e^-/Å^2$ and 1 × 10^5 $e^-/Å^2$ for tf-ADF stack (to have enough signal to see any features). All images share a normalized intensity range from 0 to 1, scaled by the maximum value of their respective 3D stack. All scale bars are 3 nm.

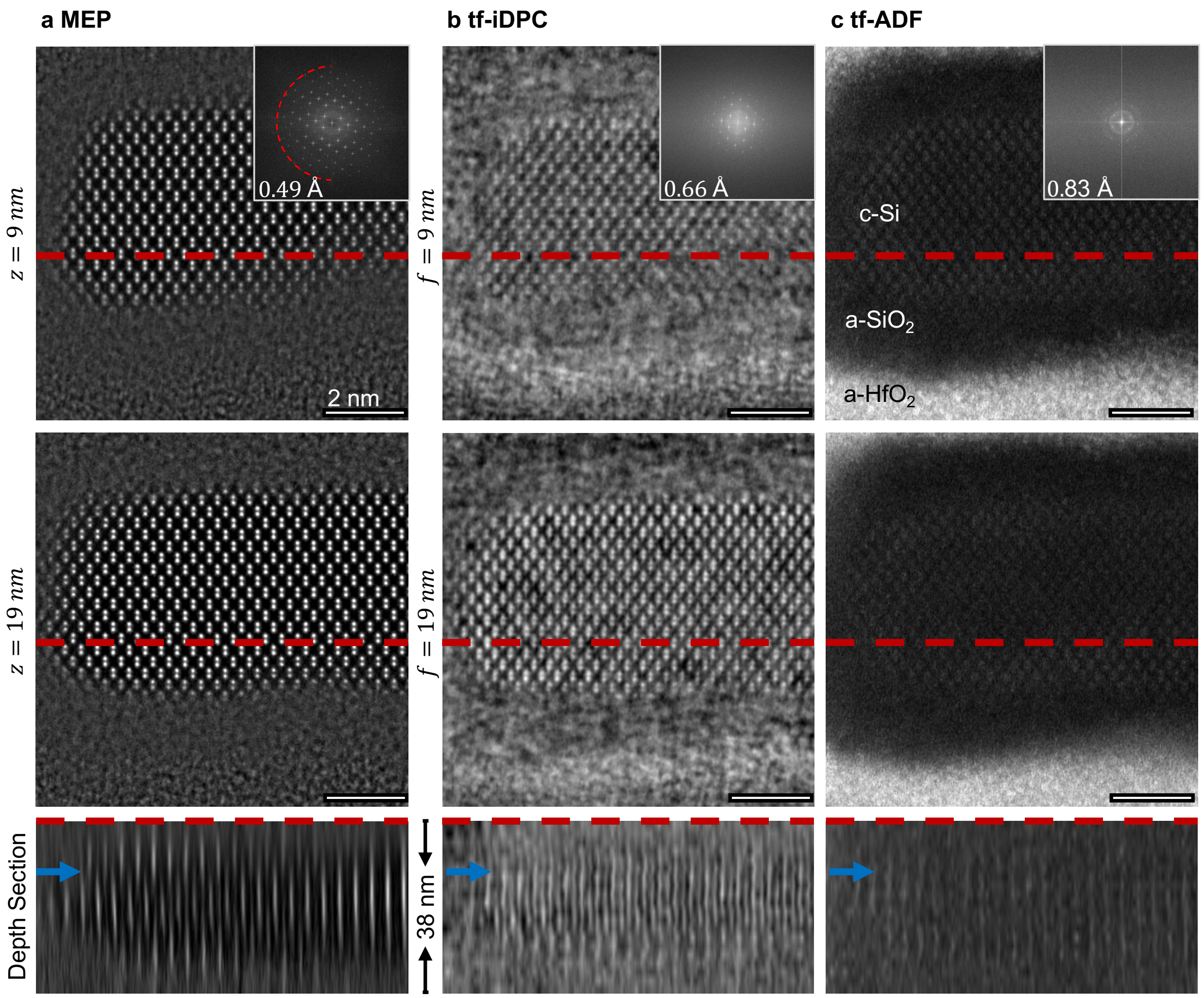

Supplementary Figure 4. Selected slices comparing experimental measurements of the different methods and 2D information transfer, with a depth section highlighting a stacking fault. a MEP, **b** tf-iDPC, and **b** tf-ADF images, each shown at two depths (9 nm and 19 nm), with a horizontal depth section (along the red dotted lines) below, illustrating stacking faults. MEP achieves higher in-plane resolution (0.49 Å) compared to tf-iDPC (0.66 Å) and tf-ADF (0.83 Å). Defects in depth reduce resolution and introduce artifacts in the through-focal imaging methods, complicating post-acquisition alignment, diminishing depth resolution, and reducing feature detection reliability. Stacking faults (blue arrows), clearly visible in MEP, are obscured in tf-iDPC and tf-ADF due to these artifacts. All images share a normalized intensity range from 0 to 1, scaled by the maximum value of their respective 3D stack. Total dose: 0.5×10^5 e$^-$/Å^2 for MEP, and 1×10^5 e$^-$/Å^2 for both tf-iDPC and tf-ADF.

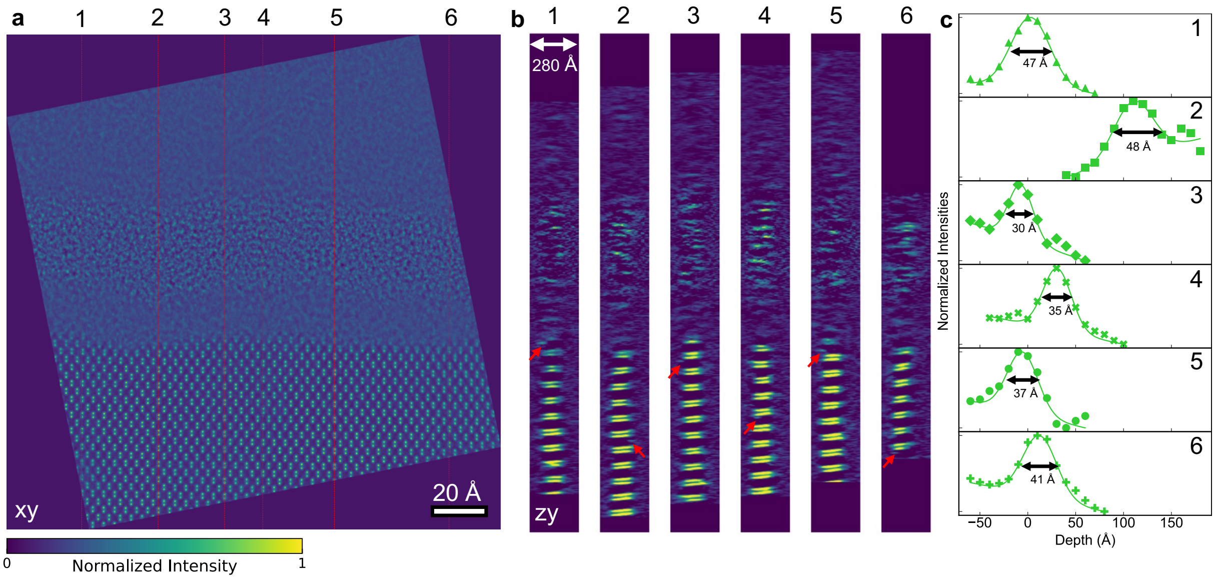

Supplementary Figure 5. Experimental estimation of MEP depth blur from isolated single-atom features. a xy projection of the MEP reconstructed atomic potential (same as Figure 5) of a planar c-Si/a-SiO_2/a-HfO_2 sample, with the numbered red dash lines indicating the positions of depth slices shown in **b**. **b** Depth-resolved intensity profiles (zy sections) from **a**, with red arrows pointing at isolated surface atoms tracked across depth. **c** For each highlighted atom (labelled 1-6 and marked with a red arrow in **a-b**), we extract a local axial intensity profile and fit it using a Gaussian peak plus a linear background term, accounting for the sloped baseline arising from surrounding amorphous contrast. The resulting full-width-at-half-maximum (FWHM) values are shown below each profile. Across all six atoms, the Gaussian+linear fits yield consistent depth blurs of 30–48 Å, corresponding to $\Delta z = 40 \pm 7$ Å (s. d.). Equivalent measurements cannot be performed for tf-ADF or tf-iDPC because finite-dose images are too noisy and individual atoms often shifted between consecutive frames. All images share a normalized intensity range from 0 to 1, scaled by the maximum value of their respective 3D stack. Image contrast has been further adjusted in the panels of **b** to highlight individual atoms for clarity. Note that the resolution and voxel size are anisotropic in panel **b.**

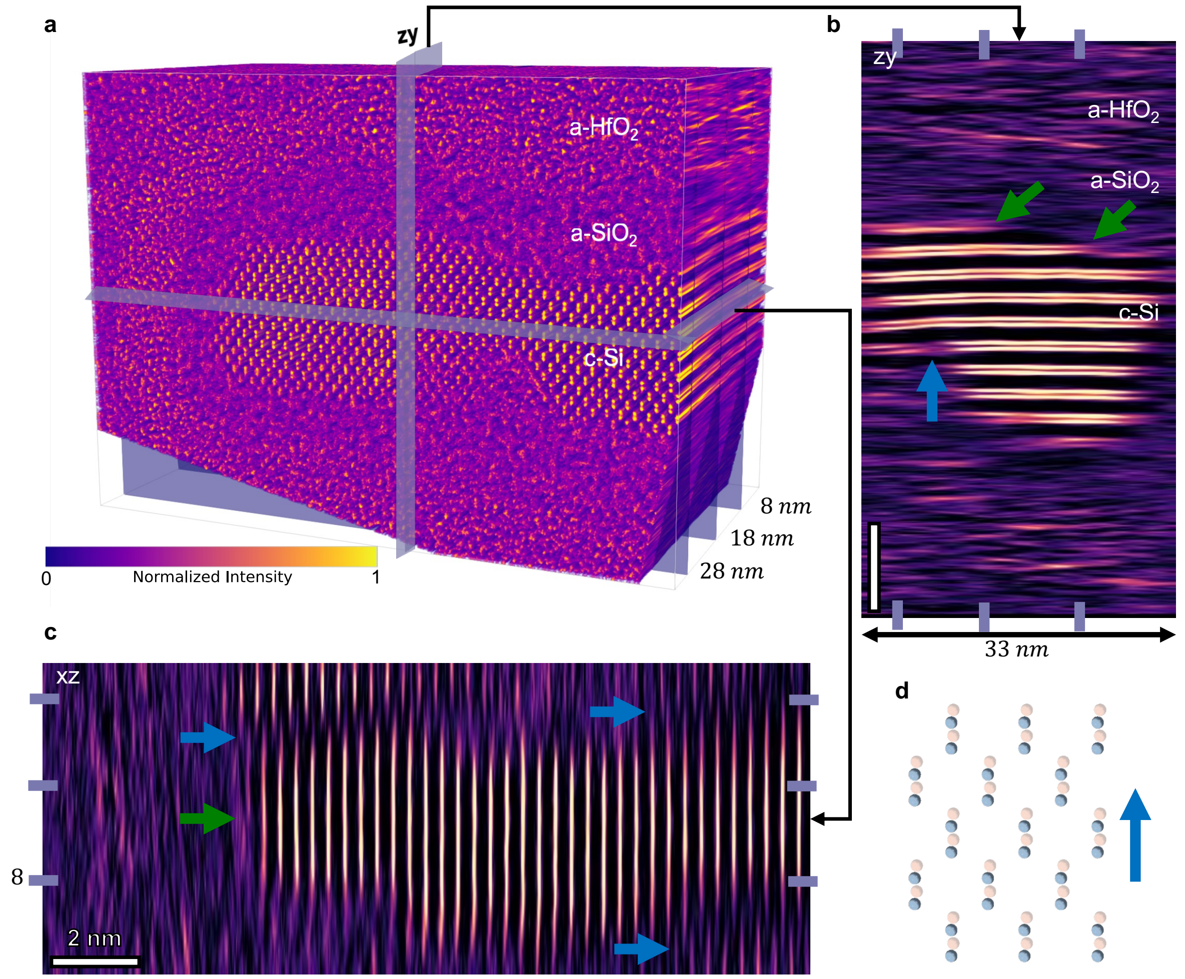

Supplementary Figure 6. MEP reconstructed cuboid from measured data on the GAA device with depth sections revealing stacking faults and step edges. a A 3D cuboid representation of the reconstructed device, illustrating all sliced planes (highlighted in green in panels **b** and **c**) with labeled components visible in three dimensions. The cuboid dimensions are 18 nm × 13 nm × 33 nm (not to scale in depth). **b** Depth section along the zy plane shown in **a**, highlighting a region with missing crystalline Si accompanied by a stacking fault (blue arrow). Step edges are visible at the c-Si/a-SiO_2 interface (green arrows), and bright streaks in the amorphous region indicate the presence of Hafnium atoms. **c** Depth section along the xz plane highlighted in **b**, showing additional stacking faults (blue arrows) and a step edge at the silicon interface (green arrow). **d** Schematic depiction of a stacking fault, illustrating a half-unit-cell mis-registry between Si layers in depth. All images share a normalized intensity range from 0 to 1, scaled by the maximum value of their respective 3D stack. Scale bars: 5 nm.

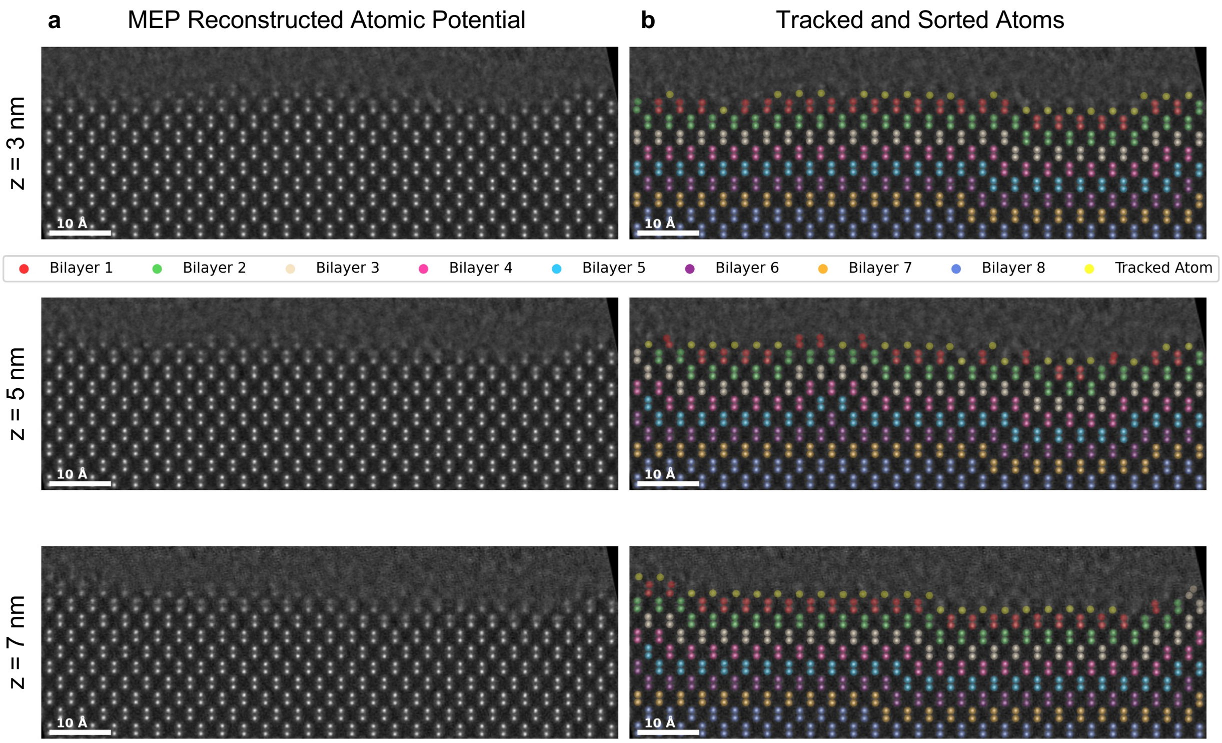

Supplementary Figure 7. Selected slices of atom tracking on the measured planar c-Si/a-SiO_2 interface. a Selected depth slices of MEP reconstructed atomic potential as a function of depth. **b** Same as **a** with superimposed tracked atoms separated into Si-Si bilayers from the interface. Tracked atoms without a nearest neighbor are colored yellow.

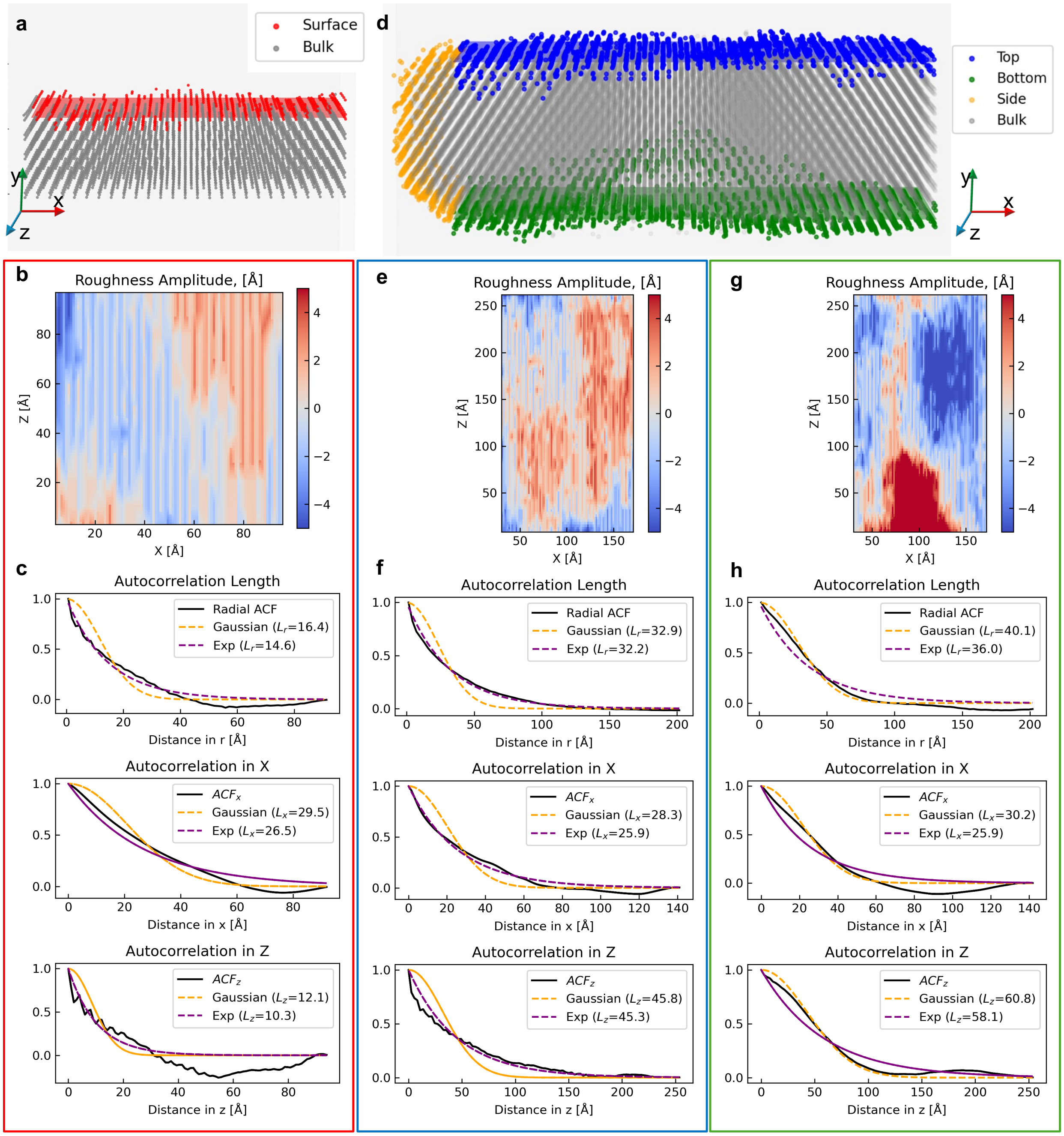

Supplementary Figure 8. Atomic-scale roughness, correlation length scales, and estimated mobility reduction of planar and GAA c-Si/a-SiO_2 interfaces. a–c Planar reference interface. **a** 3D rendering of tracked atoms: bulk atoms are shown in gray, while surface atoms and the fitted reference plane are shown in red. **b** Roughness amplitude as the residuals from the reference plane, representing local deviations form an atomically sharp interface. **c** Autocorrelation analysis of roughness: the radial autocorrelation function (ACF) (top) and line cuts along x and z (bottom). The planar interface exhibits RMS roughness of 1.7 Å and an average correlation length of 14.6 Å. The ACF decays exponentially, consistent with abrupt, step-like morphology. **d–h** GAA-2 device.

d 3D rendering of tracked atoms: bulk in gray; surface atoms and fitted facet planes colored top (blue) and bottom (green). **e–f** Residuals and ACFs for the top interface, with RMS roughness of 2.1 Å and an average correlation length of 32.2 Å, again well-described by an exponential model. **g–h** Residuals and ACFs for the bottom interface, showing RMS roughness of 3.8 Å and an average correlation length of 40.1 Å with a decay shape that deviates from the exponential model and looks more Gaussian, suggesting more complex morphology (such as the "mouse bite"), likely driven by interdiffusion and growth-related defects such as misfit dislocations (e.g. the "mouse-bite"). Across interfaces, exponential ACF models best describe smoother surfaces, while rougher ones show ambiguous, more Gaussian-like behavior. This metrology reveals critical process-dependent variations relevant for classical scaling limits. These direct measurements can clarify longstanding ambiguity in interface roughness models derived from 2D projections and enable direct measurement of buried 3D morphology.

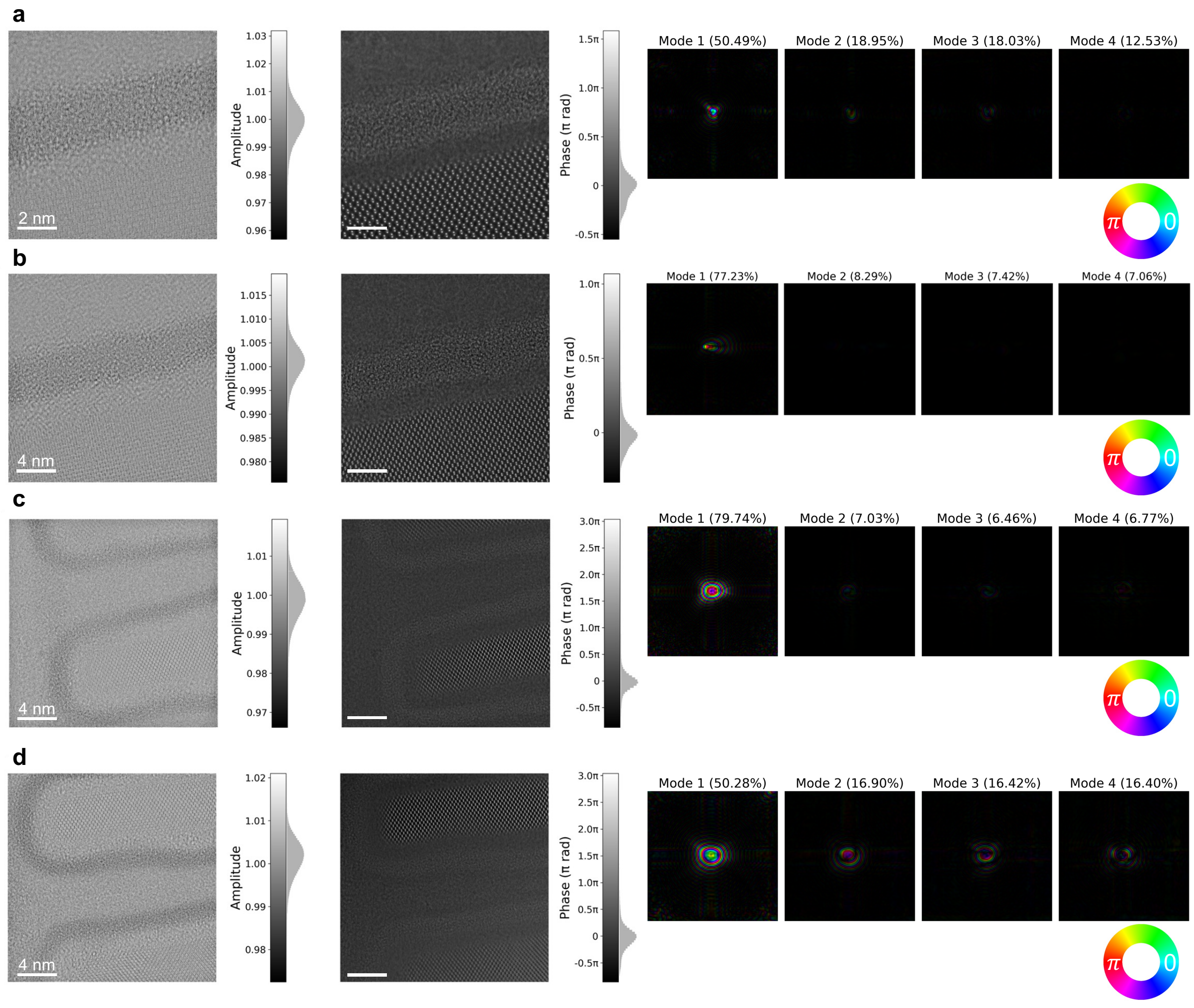

Supplementary Figure 9. MEP reconstruction summaries: a planar interface dataset 1, b planar interface dataset 2, c GAA structure 1, and d GAA structure 2. For each row, from left to right: stack-averaged amplitude, projected phase of the reconstructed object (both with corresponding color bars), and histograms showing the distributions of these values. Also shown are the reconstructed mixed-state probe amplitude (brightness) and phase (color) distributions at the entrance surface for each probe mode, along with their respective intensity percentages. The plotted object amplitude represents the average across all slices, while the projected object phase corresponds to the cumulative phase contributions from all slices. The amplitude for each slice remains close to one, as each slice behaves like a near-pure phase object. Deviations from unity are primarily due to scattering beyond the outer detector collection angle, which is more pronounced in regions containing heavier scatterers, such as hafnium (Hf). Residual aberrations, including the trifold astigmatism visible in the reconstructed probes, underscore the robustness of the MEP algorithm in recovering and separating both object and probe characteristics, even under complex experimental conditions.

Supplementary Table 1. Depth-resolution comparison for MEP and through-focal imaging. The theoretical values correspond to multislice simulation-based estimates shown in Figure 3. Experimental depth resolutions were measured from the reconstruction shown in Figure 5 and Supplementary Figure 5**.** Through-focal ADF and iDPC did not yield experimental depth-resolution estimates because the through-focal series were too noisy to localize individual atoms reliably.

	Theory	Experiment
MEP	22	40 ± 7 Å
tf-ADF	40	-
tf-iDPC	57	-

Supplementary Table 2. Table of measured roughness and mobility estimates. The table summarizes RMS roughness (Δ) and in-plane correlation length (Λ) for planar and GAA interfaces, along with effective relative mobility (μ/μ_{planar}) estimated via the long-wavelength approximation, $\mu \propto 1/(\Delta^2\Lambda^2)$, assuming all other parameters equal for all interfaces. This relationship illustrates the strong mobility reduction for rougher interfaces: taking the the planar interface as a reference (relative mobility of 100 %), the long-wavelength mobilities of GAA top and bottom interfaces would reduce to 16.4 % and 2.8 %, respectively.

	Planar (a-c)	GAA Top (d-f)	GAA Bottom (d,g-h)
RMS roughness (Δ), (Å)	1.7	2.1	3.8
Correlation length (Λ), (Å)	14.6	32.2	40.1
Effective Relative Mobility, (%)	100	13.5	2.7

Supplementary Table 3. Experimental Parameters for Multislice Electron Ptychography (MEP) and Through-Focal Imaging

Parameter	Planar Interface (Supp. Fig. 1a)	Planar Interface 2 (Supp. Fig. 1b)	GAA-1 (Supp. Fig. 1c)	GAA-2 (Supp. Fig. 1d)	tf-ADF (GAA-2)	tf-iDPC (GAA-2)
Microscope	Cs-corrected Thermo Fisher Spectra 300 X-CFEG					
Accelerating voltage, (kV)	300					
Detector	EMPAD-G2 (4D-STEM)				Thermo Fisher ADF	Thermo Fisher Panther (8-section DPC)
Specimen thickness, (Å) [simulation size for MEP]	250	220	400	380	400	
Probe convergence semi-angle (α, mrad) [clibration used for MEP]	29.4	30.24	29.78	30.04	30	
Scan step size, (Å)	0.432	0.58	0.82	0.81	0.2	
Scan dwell time, (μs)	100				0.6	
Scan size, (px)	256 × 256				1024 × 1024	
Field of view, (Å)	110.6	148.5	210	207	210	
Detector collection range, (mrad)	53	53	42	53	40–250	9–34
Total dose, (e-/Å2)	1.8×10^5	1×10^5	0.5×10^5		1×10^5	
Defocus, (Å) [calibration used for MEP]	–80	–50	–106	–60	-	
In-plane rotation, (°)	–90	–90	0		-	
x, y tilts, (mrad)	–2.0, –1.6	0, 0	0, 0		-	
Measured radius of bright field disk, (px)	36	36	46	36	-	
Diffraction sampling (mrad per px)	0.82	0.84	0.65	0.83	-	
Number of probe modes	4				-	
MEP slice thickness, (Å)	1				-	
Layer regularization	0.5				-	
Number of focal planes	-				20	
Focal series step size, (Å)	-				20	

Supplementary Table 4. Simulation Parameters for MEP, tf-ADF, and tf-iDPC Comparisons

Parameter	Value / Description
Simulated systems	c-Si / a-SiO_2 / a-HfO_2 interface (66 Å thick) and pMOS GAA structure (200 Å thick)
Acceleration voltage	300 keV
Probe semi-convergence angle (α)	30 mrad
Aberrations applied	Spherical (Cs = 1 μm), Chromatic (Cc = 2.46 mm)
Energy spread	0.4 eV
Chromatic broadening	$dE/E_0 \cdot Cc \approx 3.28$ nm
Total electron dose	Interface: 2.5 × $1e^5$ $e^-/Å^2$ (total dose for each mode)<br>pMOS GAA: 0.4 × 10^5 $e^-/Å^2$ for MEP and tf-iDPC; 1 × 10^5 $e^-/Å^2$ for tf-ADF
Noise model	Poisson noise added to diffraction patterns
Scan step size (real space)	tf-iDPC / tf-ADF: 0.14 Å; MEP (c-Si/a-SiO_2): 0.44 Å; MEP (GAA): 0.75 Å
Through-focal parameters	Focal step size: 10 Å; number of focal planes: thickness/10 Å + 3-4 extra planes on each side of the sample
Annular detector ranges	iDPC: bright-disk, 0-30 mrad; ADF: 60–150 mrad
Pixelated detector sampling	128 × 128 px diffraction patterns; MEP max collection angles: 60 mrad (2α) or 90 mrad (3α)
MEP reconstruction parameters	Slice thickness: 5-10 Å; layer regularization: 0.1; defocus: -100 Å; number of probe modes: 4
