# Supplementary material for: 3D atomic-scale metrology of strain relaxation and roughness in Gate-All-Around transistors via electron ptychography
Source: Nat Commun. 2026 Feb 23;17:3561. doi: 10.1038/s41467-026-69733-1 (PMC13087028; doi:10.1038/s41467-026-69733-1)
Supplement: Supplementary file 2 — Description of Additional Supplementary Files [file 41467_2026_69733_MOESM2_ESM.pdf]

Description of Additional Supplementary Files

Supplementary Movie 1: Reconstructed 3D potential of the planar c-Si/a-SiO2 interface. **a.** Multislice Electron Ptychography (MEP) reconstructed electrostatic potential visualized as a function of depth. **b.** Same as a, with tracked atomic positions overlaid and grouped into bilayers from the interface.

Supplementary Movie 2: Reconstructed 3D potential of GAA Device 1*.* **a.** MEP reconstructed electrostatic potential as a function of depth. **b.** Same as a, with tracked atoms overlaid and organized into bilayers from the gate-channel interface.

Supplementary Movie 3: Reconstructed 3D potential of GAA Device 2. **a.** MEP reconstructed electrostatic potential as a function of depth. **b.** Same as **a,** with tracked atoms overlaid and separated into bilayers from the interface.

Supplementary Movie 4: 3D visualization of the silicon channel geometry in GAA Device 1. The reconstructed shape of the crystalline silicon channel is shown in 3D, highlighting variations in cross-sectional geometry and showing the various missing crystalline regions.

Supplementary Movie 5: 3D visualization of the silicon channel geometry in GAA Device 2*.* Same as Movie 4, but for GAA Device 2, which has more regular shape in comparison.

Supplementary Movie 6: Summary of MEP reconstruction of GAA Device 1*.* Sequential cross-sectional views through the 3D MEP reconstruction, highlighting the crystalline silicon channel, the “mouse-bite”, and its extracted surface geometry.
